# Association between Subclinical Malaria Infection and Inflammatory Host Response in a Pre-Elimination Setting

**DOI:** 10.1371/journal.pone.0158656

**Published:** 2016-07-07

**Authors:** Thomas J. Peto, Rupam Tripura, Sue J. Lee, Thomas Althaus, Susanna Dunachie, Chea Nguon, Mehul Dhorda, Cholrawee Promnarate, Jeremy Chalk, Mallika Imwong, Lorenz von Seidlein, Nicholas P. Day, Arjen M. Dondorp, Nicholas J. White, Yoel Lubell

**Affiliations:** 1 Mahidol-Oxford Tropical Medicine Research Unit, Faculty of Tropical Medicine, Mahidol University, Bangkok, Thailand; 2 Centre for Tropical Medicine and Global Health, University of Oxford, Oxford, United Kingdom; 3 National Center for Parasitology, Entomology and Malaria Control, Phnom Penh, Cambodia; 4 Worldwide Antimalarial Resistance Network, Faculty of Tropical Medicine, Mahidol University, Bangkok, Thailand; 5 Department of Molecular Tropical Medicine and Genetics, Faculty of Tropical Medicine, Mahidol University, Bangkok, Thailand; Institut de Recherche pour le Développement, FRANCE

## Abstract

**Background:**

Subclinical infections in endemic areas of Southeast Asia sustain malaria transmission. These asymptomatic infections might sustain immunity against clinical malaria and have been considered benign for the host, but if they are associated with chronic low-grade inflammation this could be harmful. We conducted a case-control study to explore the association between subclinical malaria and C-reactive protein (CRP), an established biomarker of inflammation.

**Methods:**

Blood samples from asymptomatic villagers in Pailin, Western Cambodia were tested for malaria by high-volume ultra-sensitive polymerase chain reaction (uPCR) to determine the *Plasmodium* species. Plasma CRP concentration was measured in 328 individuals with parasitaemia (cases) and compared with: i) the same individual’s value at the first time point when they had no detectable parasites (n = 282); and ii) age- sex- and village-matched controls (n = 328) free of *Plasmodium* infection. Plasma CRP concentrations were compared against thresholds of 3mg/L and 10mg/L. Subgroup analysis was carried out for cases with *P vivax* and *P falciparum* mono-infections.

**Results:**

Median plasma CRP level for all samples was 0.59mg/L (interquartile range: 0.24–1.64mg/L). CRP concentrations were higher in parasitaemic individuals compared with same-person-controls (p = 0.050); and matched-controls (p = 0.025). 4.9% of samples had CRP concentrations above 10mg/L and 14.6% were above 3mg/L. Cases were more likely to have plasma CRP concentrations above these thresholds than age/sex matched controls, odds ratio 3.5 (95%CI 1.5–9.8) and 1.8 (95%CI 1.1–2.9), respectively. Amongst cases, parasite density and CRP were positively correlated (p<0.001), an association that remained significant when controlling for age and fever. Individuals with *P*.*vivax* mono-infections had the highest plasma CRP concentrations with the greatest association with parasitaemia.

**Discussion:**

In this setting persistent malaria infections in asymptomatic individuals were associated with moderately elevated plasma CRP concentrations; chiefly evident in cases with *P*.*vivax* mono-infections. As well as interrupting malaria transmission within the community, treatment of asymptomatic malaria infections, in particular radical cure of vivax malaria, may benefit the health of infected individuals.

## Background

A subclinical reservoir of malaria plays an important role in sustaining transmission in areas of seasonal malaria and presents an obstacle to malaria elimination [1]. The size, dynamics, and characteristics of this reservoir are still poorly understood [2, 3]. From an evolutionary perspective, an optimal strategy in low transmission settings is for malaria parasites to persist at a subclinical level within a host [4]. The optimum density would produce sufficient gametocytes to allow transmission, but be below the level at which clinical symptoms lead to either clearance by activation of the immune system, or the seeking of treatment; either of which result in the end of the infection [5].

The effects of submicroscopic parasitaemia on the host beyond the risk of developing clinical malaria and contributing to splenomegaly and anaemia are not well-understood, as these infections are below the level of detection by most standard methods. A recent review of the potential burden of subclinical infections beyond clinical cases, implicated these ‘chronic infections’ with higher risk of susceptibility to bacteraemias, anaemia, and all-cause mortality, but the underlying biology and causal pathways are not clear [6]. Whether subclinical infections induce an inflammatory response, for instance, has been studied in only a few places [7–9] and only once in a low transmission setting. It is of potential pathological importance as low levels of inflammation are known contributors to leading causes of morbidity including myocardial infarction, stroke, diabetes, and metabolic syndrome, as well as an increased risk of overall mortality [10].

If subclinical malaria infection is associated with an inflammatory response this could contribute to similar morbidities, particularly if these persist for prolonged periods. It is also important to better understand if such associations are more or less evident in different *Plasmodium* species, as this could inform considerations into either radical cure of *P*.*vivax* infections, and or wide-spread treatment of otherwise healthy individuals with ACTs to help achieve *P*.*falciparum* elimination.

C-reactive protein (CRP) is a cytokine-induced acute phase protein and biomarker of inflammation and infection. CRP plays a role in host defence by activating the complement pathway and as a pro-inflammatory mediator. The median Plasma CRP concentrations in the population is 1.5mg/L with an interquartile range of 0.3–3.5mg/L, a range that is stable across sex and ethnic groups. CRP concentrations increase markedly during acute inflammatory events [11, 12] notably acute bacterial infections and systemic protozoal infections such as malaria. Elevated CRP is also widely used as a predictor of cardiovascular disease with a threshold of 3mg/L used to define individuals at high risk [13, 14]. In addition to cardiovascular disease, CRP has been shown to be associated with a range of other morbidities including diabetes and cancer, and has also been shown to predict all-cause mortality [15].

A number of studies have reported a relationship between clinical malaria and CRP [16–18]. Anti-disease immunity that controls parasite densities and illness might suppress the inflammatory response to the infection, but this relationship has not been assessed in detail. If subclinical malaria parasitaemia is associated with chronic inflammation this could justify strategies to treat these infections, given the health risks to the individual of persistently raised inflammatory proteins [19–21]. Data are available from Ghana showing that in high transmission settings (prevalence>85% PCR detected plasmodium infections in apparently healthy volunteers aged 8–95), CRP levels were positively associated with *P falciparum* infection [8]. A recent study in Indonesia investigated possible haematological, vascular and inflammatory effects of asymptomatic infection and found evidence of all three in children, with CRP being elevated in both children and adults [9]. There were insufficient *P*. *vivax* cases to determine whether there were differences between *Plasmodium* species.

A better understanding of plasma CRP concentrations in individuals in malaria endemic settings is needed to determine the potential use of CRP as a guide for antibiotics in febrile patients with negative malaria tests. In a recent study CRP was found to be a useful biomarker for distinguishing between viral and bacterial infections in febrile patients in the Mekong [17]. Treatment algorithms based on CRP and malaria RDTs have the potential to guide the rational use of antibiotic treatment, but if plasma CRP concentrations in these populations are commonly elevated for other reasons this could undermine the utility of such an approach.

This study therefore addresses the following questions: i) what is the distribution of plasma CRP concentrations in apparently healthy individuals in rural Southeast Asian settings? ii) are plasma CRP concentrations raised in asymptomatic parasitaemic individuals, and are plasma CRP concentrations correlated with parasite loads? iii) is there evidence of a difference in CRP concentrations in individuals with *P*.*vivax as compared with P*.*falciparum* subclinical infections?

## Results

Of 2151 individual villagers for whom uPCR results were available, 328 (15.2%) were found to be positive for malaria parasitaemia in at least one instance with a corresponding plasma sample available for CRP testing (Table 1). *P*.*falciparum* and *P*.*vivax* mono-infections were identified in 12% and 35% of these 328 cases, respectively. The remaining cases had either a *Plasmodium* infection with indeterminate species (51.5%) or a mixed infection (1.5%). All 328 cases were assigned a matched control, surveyed in the same month. In 282 cases a sample without detectable parasitaemia and with a CRP result was available to act as a same-person control.

**Table 1:** Demographic data for same person controls, age- sex- village matched controls and cases, and those for the sub-groups with *P*.*falciparum* and *P*.*vivax* mono-infections.

	ControlNext -ve result in the same person	ControlAge- village- and sex-matched	CaseMalaria parasites of any species	Cases with *P*. *vivax* mono-infection	Cases with *P*. *falciparum* mono-infection
*n (% of all infections)*	282	328	328	115 (35%)	40 (12%)
Mean age (SD) (years)	(same as case)	25.6 (17)	26.35 (17)	28 (16)	28 (18)
% Male (n)	(same as case)	210 (64%)	210 (64%)	81 (70%)	23 (57%)
No. with fever (%)	31 (10.9%)*	5 (1.5%)**	44 (13.3%)	20 (17.4%)	5 (12.5%)
Mean tympanic temperature (SD), degrees C	37.0 (0.40)	36.9 (0.33)	37.1 (0.50)	37.1 (0.60)	37.2 (0.30)
Recent history of illness	33 (11.6%)	81 (24.6%)	85 (25.8%)	22 (19%)	16 (40%)
Geometric mean parasitaemia (95% CI) /mL	NA	NA	2178 (1444, 3285)	35036 (17644–69567)	9117 (2839–29279)

* p = 0.362

** p < 0.001

### Differences in distribution of plasma CRP concentrations

**Fig 1:**
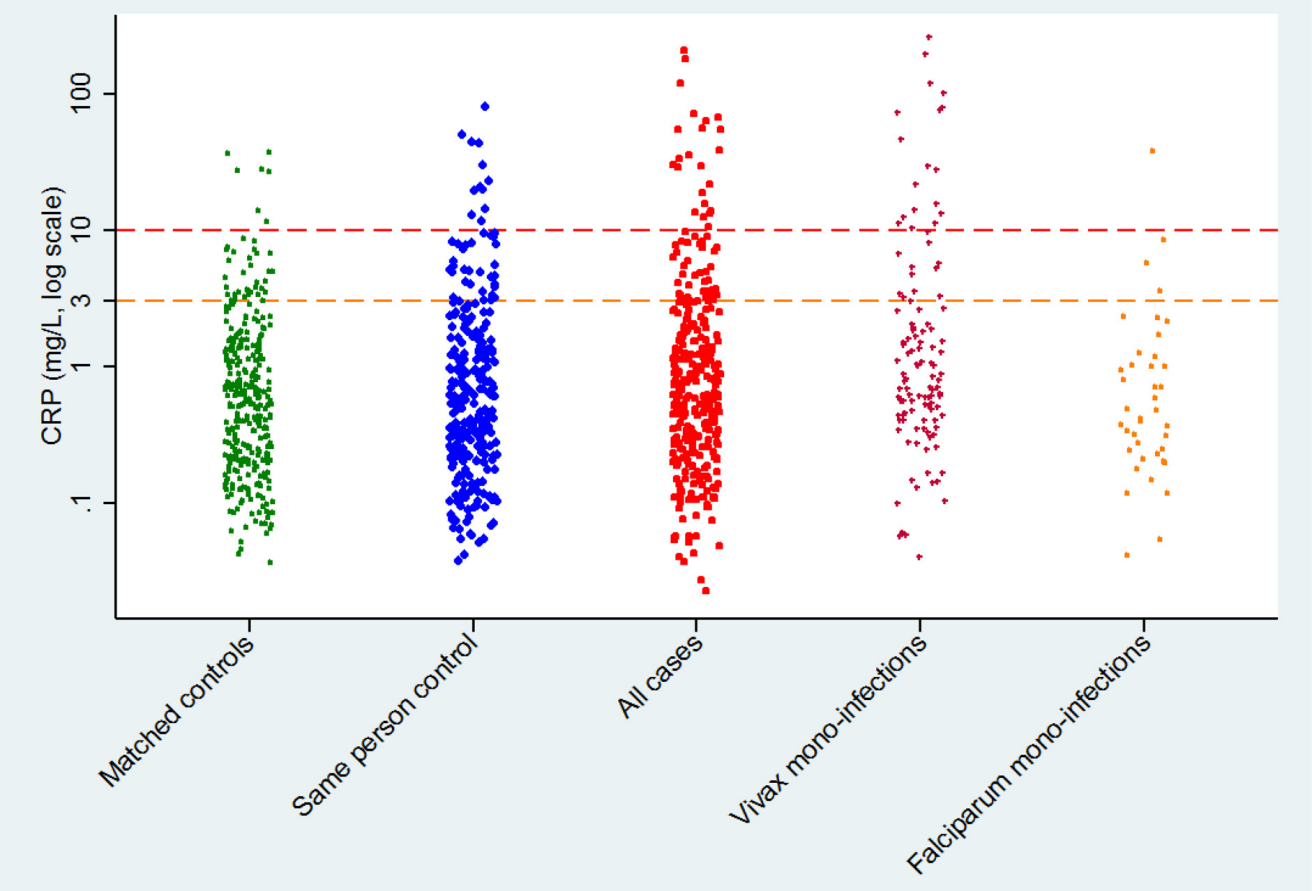
Distribution of CRP concentrations in both sets of controls, cases, and in cases with vivax and falciparum mono-infections.

The median plasma CRP level for all samples was 0.59mg/L with an interquartile range of 0.24–1.64mg/L and a full range of 0.02-263mg/L; the data are shown in Fig 1. Plasma CRP concentrations were higher in parasitaemic individuals compared to matched-controls (p = 0.025) and to same-person-controls (p = 0.050), with a median value of 0.66mg/L in cases, 0.52mg/L in age/sex matched controls, and 0.58mg/L in same-person controls; Table 2 and Fig 1.

**Table 2:** CRP values and distributions in cases and controls.

Plasma CRPconcentrations	ControlNext–ve result in the same person (n = 282)	Control Age- village- and sex-matched (n = 328)	CaseMalariaparasites of any species (n = 328)	Cases with *P*. *vivax* mono-infection (n = 115)	Cases with *P*. *falciparum* mono-infection (n = 40)
Median CRP mg/L (IQR; range)	0.52 (0.24, 1.67; 0.05, 98.3)*	0.58 (0.21, 1.4; 0.04, 45.2)**	0.66 (0.27, 1.99; 0.02 263.1)	0.89 (0.44–3.31; 0.04–263)^‡^	0.43 (0.22–1.13; 0.05–36.9) ^‡‡^
Number (%) of subjects with CRP>3mg/L	46 (16.3%)	34 (10.4%)	57 (17.3%)	29 (25.2%)	4 (10%)
Number (%) of subjects with CRP>10mg/L	14 (5.0%)	7 (2.1%)	25 (7.6%)	18 (15.7%)	1 (2.5%)

*p = 0.0497 for difference between cases and same person controls

**p = 0.0248 for difference between cases and same age- sex- matched controls

^‡^p = 0.011 for difference between *P*.*vivax* mono-infection cases and their same age- sex- matched controls and p = 0.004 for the same-person control samples.

^‡‡^p = 0.25 for difference between *P*.*falciparum* mono-infection cases and their same age- sex- matched controls and p = 0.41 for the same-person control samples.

With the age/sex matched controls this difference remained significant when excluding individuals who reported an episode of illness in the previous 48 hours (p = 0.022) but not when excluding individuals with a fever at the time of the survey (while self-reporting as being asymptomatic) (p = 0.103). In the same-person control comparison the differences were not significant once febrile individuals or those with a reported episode of illness were excluded from the analysis.

Plasma CRP concentrations were highest in cases with *P*.*vivax* infections, with a median of 0.9mg/L, also significantly higher than their matched and same person controls (p = 0.011 and 0.004 respectively). The differences remained significant also with the exclusion of cases with a reported illness in the previous 48 hours and in those with a fever at the time of the survey (p = 0.018 and 0.014, respectively). There was no significant difference in plasma CRP concentrations between cases with a mono *P*.*falciparum* infection and those of either set of controls.

### Plasma CRP concentrations in relation to clinical thresholds

In aggregate, 14.6% of asymptomatic villagers had plasma CRP concentrations above a threshold of 3mg/L, and 4.9% had plasma CRP concentrations above a threshold of 10mg/L. There was a significant difference between cases and controls (Table 2), with 17% of cases having plasma CRP concentrations above the 3 mg/L threshold as compared with 10% in the age/sex matched controls (odds ratio 1.8, 95%CI 1.1–2.9). In the same-person controls the proportion of individuals with elevated plasma CRP above the threshold was similar to that in the cases, 16% (odds ratio 1.1, 95% CI 0.7–1.8).

A similar difference was found with the higher threshold of 10mg/L (Table 2), with 7.6% of cases having a CRP reading above the threshold as compared with 2.1% in the age/sex matched controls (p<0.001) and 5% in the same-person controls (p = 0.14).

In the subgroups of patients with mono-infections, *P*.*vivax* cases were more likely to have plasma CRP concentrations above the threshold of 3 mg/L as compared with the age-sex matched controls (25.2%, with an OR of 2.7 (95% CI 1.3–6.3)). Likewise the OR for plasma CRP concentrations being above a threshold of 10mg/L was 9 in the vivax cases as compared with their age-sex matched controls (95% CI 2.2–80). No differences were found in the odds of having elevated CRP levels above the thresholds in cases with falciparum mono-infections as compared with either set of controls (both p>0.3).

### Parasitaemia and CRP

**Fig 2:**
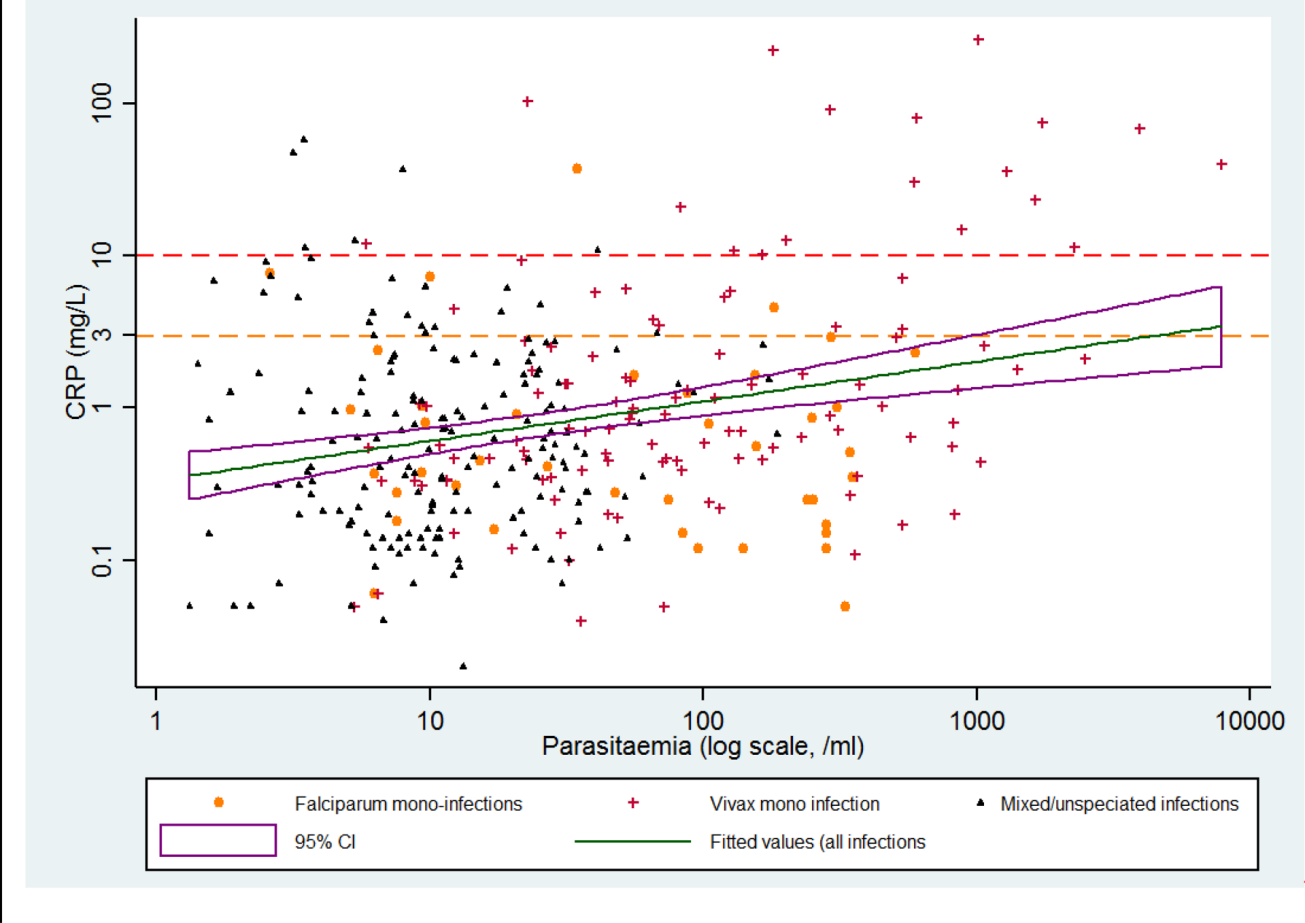
Scatterplot and linear prediction for the association between parasitaemia and plasma CRP concentrations.

The geometric mean parasitaemia (95% CI) in cases was 2178/mL (1444–3285), Table 1. There was a positive correlation between log parasitaemia and log plasma CRP (Pearson’s correlation coefficient = 0.26, p <0.001); this result was unchanged by the exclusion of cases with fever (correlation coefficient = 0.25, p <0.001). When including only cases with a vivax mono-infection the correlation coefficient increased to 0.44 and remained statistically significant (p<0.001), despite the lower number of participants. In a linear regression model, while fever was itself significantly associated with elevated CRP (p = 0.013), the association between parasite count and plasma CRP remained significant after controlling for fever (p<0.001) and remained positive when adjusted for age. The data for log transformed parasite counts and CRP concentrations and their linear prediction are shown in Fig 2.

## Discussion

Subclinical malaria infections could have a significant health burden beyond that posed by clinical cases [6]. This study found plasma C-reactive protein (CRP) concentrations in individuals with subclinical submicroscopic malaria infection to be raised as compared with age/sex matched controls, as well as in the same individuals reassessed when they did not have detectable parasitaemia. The differences were mostly of small magnitude but may have a clinical significance in prolonged infections, particularly in *P*. *vivax*, where the differences were most pronounced, and due to the persistent nature of the infection.

The uPCR method used in this study has a lower limit of detection of 22 parasites/mL which detects nearly 75% of *P*.*falciparum* infections and over 85% of *P*.*vivax* infections [5]. There was a significant association between parasitaemia and plasma CRP concentrations, which remained significant when adjusting for fever. The association was particularly strong in cases with a vivax mono-infection, and less apparent in those with a falciparum infection, although the latter constituted only a small minority of cases (12%). The higher plasma CRP concentrations in vivax malaria might be explained by a heightened inflammatory host response as compared with falciparum malaria [22].

Individuals with acute illness were excluded by design from the malaria survey but some participants were found to have a fever and/or reported an episode of illness in the last 48 hours, and some of the differences in plasma CRP concentrations between cases and both groups of controls were no longer significant once these individuals were excluded from the analysis. A degree of interdependence between these inflammatory responses to parasitaemia is expected. The frequent presence of fever and/or elevated CRP in individuals with detectable parasites in a community setting supports the notion that what are often referred to as submicroscopic, ‘asymptomatic infections’ could in fact have a detrimental health impact, even if they do not result in health seeking behaviour.

There was a higher prevalence of elevated plasma CRP and a higher proportion of febrile individuals in same-person controls as compared with the age/sex matched controls. This might be explained by persisting infections that were not detected by uPCR. There are, however, other potential factors that could explain these differences that were not accounted for in the matching, such as forest goers that could be at greater risk of other non-malarial fevers, as well as other genetic or behavioural predispositions for elevated CRP.

Regarding the use of CRP to guide the use of antibiotics in febrile patients with a negative malaria test, this study confirms that plasma CRP concentrations in this rural, Southeast Asian population were above a conservative threshold of 10mg/L in only 4.6% of individuals. This threshold is in fact probably lower than is likely to be used in this context; a recent study identified 20mg/L as a potential threshold [17], in which case only 2.9% in this study population would have higher values than this threshold. This implies that CRP guided treatment in febrile patients would not be hindered by a large proportion of ‘false positive’ tests due to high prevalence of elevated CRP in the general population.

### Limitations

While uPCR is the most sensitive test available for these surveys, some infections are still undetectable, and some of the controls, particularly in the same-person control group could in fact be infected. While this might result in smaller differences in plasma CRP concentrations between the groups this would only strengthen the association between parasitaemia and elevated CRP. Second, there is a possibility of confounders and reverse causality, whereby for instance other causes of fever or elevated CRP might trigger relapse of malaria parasites. Larger studies with repeated CRP measurement before and after infection would be needed to explore this. Other possible risk factors and confounders that could be implicated in elevated CRP and/or parasitaemia would need to be included in the matching process. Third, the thresholds of 3mg/L and 10mg/L used to classify clinically significant elevated CRP concentrations are not well defined, although the overall finding of higher risk of elevated CRP in cases versus controls is a consistent one irrespective of the specific chosen threshold. Fourth, and potentially the most important limitation, was the inability to determine the species of 52% of all plasmodium infections, which may introduce bias into the analysis. Species determination was chiefly unavailable from low-density parasitaemias. As parasitaemia was associated with species the unavailability of results may be non-differential and a potential cause of bias.

### Conclusions

This study finds that subclinical submicroscopic malaria infection is associated with elevated plasma CRP concentrations, particularly in *P*.*vivax* infections. Prolonged inflammation and elevated plasma CRP concentrations have been implicated with cardiovascular disease and other common causes of morbidity. If these findings are verified in future studies, this would lend further weight to the treatment of subclinical infected individuals for their own health benefit, as well as for the community benefits associated with interrupting malaria transmission. The findings also reinforce the importance of radical cure of vivax malaria to avoid recurrent inflammation during repeated blood stages, even if these do not always manifest in clinical episodes.

## Materials and Methods

### Ethical approval

Ethical approval for the survey was obtained from the Cambodian National Ethics Committee for Health Research (0029 NECHR), the Oxford Tropical Research Ethics Committee (OXTREC; 1015–13), and the study was registered on clinicaltrials.gov (NCT01872702). The informed consent form allowed for use of the stored samples for further investigations to characterise malaria infections. We obtained written informed consent from all individuals or from the parent or guardian of children.

### The Study Site

Pailin is an agricultural province on the Thai-Cambodian border to the north of the Cardamom Mountains. As elsewhere in Cambodia, Pailin has seen a marked reduction in the incidence of malaria over the past decade [23]. Several factors may contribute to this decrease: the village malaria worker (VMW) programme which provides early diagnosis and treatment and records cases [24], the restriction of antimalarial drug prescription by the private sector and prohibition of artemisinin monotherapies [25], increased usage of long-lasting insecticide treated bed-nets [26], better health education [27], and environmental changes, chiefly the clearing of forested areas [28].

The three villages with the highest malaria incidence from the Pailin provincial health department’s 2012 records were selected for the study. A study malaria team was formed in each village. Meetings were held to mobilize the community to participate and inform the villagers about the study. Before enrolment individual informed consent was obtained from all participants or their guardian. The study population were self-reportedly healthy individuals; people who were acutely sick were not asked to provide blood during surveys.

### Survey methods

The survey has been reported previously [1]. In brief, over thirteen months in 2013–14 cross-sectional surveys were conducted in June, October, January, April, and June in three villages in Pailin, western Cambodia. The *de facto* population, including migrants and visitors was invited to participate in the survey and coverage averaged 80%. Those with malaria parasitaemia in the June 2013 survey were thereafter tested monthly for malaria. During surveys tympanic temperature, height, weight, and health status and health history data were collected. A 3ml blood sample was collected by venepuncture from all participants aged 5 years and above and a 0.5ml venous sample from those aged 6 months up to 5 years. Samples were stored in EDTA tubes on ice and transported daily to a hospital laboratory for processing.

### Molecular laboratory procedures

An ultra-sensitive quantitative polymerase chain reaction (uPCR) technique was used to detect malaria parasites. Validation and performance characteristics have been reported elsewhere [29]. Parasite density was estimated by Rotor-Gene Q software v 2.0.2. This measure of the number of parasite genomes was used to approximate parasite density [1, 30]. Nested PCR was used to determine the *Plasmodium* species [31]. Samples with insufficient DNA to amplify by nested PCR to determine the species were reported as *Plasmodium* species (*P*. *species*). All laboratory tests were blinded to participant malaria and health status.

### C-reactive protein assays

20ul plasma samples were thawed, split, and tested for CRP by an independent reference laboratory (Professional Laboratory Management Corp Co., Ltd, Bangkok, Thailand, ISO/IEC 15189 accredited; http://www.prolab.co.th/en/quality-assurance) using a solid phase sandwich enzyme-linked immunosorbent assay (ELISA) with conjugated detection antibody. CRP tests were performed independently by laboratory staff blinded to all participant data and their case-control status.

### Definition of cases and controls

Cases were defined as villagers who were positive for *Plasmodium* parasitaemia in at least one survey; CRP was measured in the first instance they tested positive. The first available sample with a negative result and CRP measurement was assigned as a same-person Control. All cases were also assigned an age- (within 5 years) sex- village- matched control (uPCR negative for parasites) surveyed in the same month.

### Statistical analysis and data management

The study used a cross-over design where each malaria patient served as their own control at the next time seen without malaria, as well as having age and sex matched controls from the same village. The associations between parasitaemia and plasma CRP concentrations were compared between cases and controls using the Wilcoxon matched-pairs signed rank test, for each of the control groups separately. The analysis was repeated with the exclusion of cases and controls who reported illness in the previous 48 hours and with the exclusion of febrile individuals at the time of the survey (in whom plasma CRP concentrations might be elevated for other reasons). We then compared the proportions of cases and controls with CRP concentrations that exceeded thresholds of 3mg/L and 10mg/L as predictors of cardiovascular disease [14] and infection, respectively [11]. We calculated and compared the odds ratio of having CRP concentrations above these thresholds using McNemar's chi-squared test.

The parasite counts and plasma CRP concentrations were log transformed and the correlations between them were estimated (Pearson’s test for correlation); this estimation was repeated omitting cases with fever. A linear regression model was used to graph this association, as well as the association between the age/sex matched controls and their respective plasma CRP concentrations, controlling for the presence of fever.

These analyses were then repeated for *P falciparum* and *P vivax* cases in isolation, excluding mixed infections and those where a species could not be identified.

Data were entered into Open Data Kit (ODK) on smartphones before being exported into Open Clinica. Analysis was done in STATA 14.0.
